# Supplementary material for: Assessing Feeding Difficulties in Children Presenting with Non-IgE-Mediated Gastrointestinal Food Allergies—A Commonly Reported Problem
Source: Nutrients. 2024 May 22;16(11):1563. doi: 10.3390/nu16111563 (PMC11173616; doi:10.3390/nu16111563)
Supplement: Supplementary file 1 [file nutrients-16-01563-s001.zip › nutrients-2974324-supplementary.pdf]

Supplementary Table S1

Non-IgE mediated Symptom Monitoring Questionnaire before and after the Elimination Diet

Symptoms: please indicate which symptoms your child experiences?								
	**Please indicate the severity of the symptoms:**						What age did it start:	Score at follow up:
	none	mild		moderate		severe		
**Diarrhoea:** (loose, watery, frequent stools)	0	1	2	3	4	5		
**Constipation:** (excessive straining, low frequency, hard stools)	0	1	2	3	4	5		
**Vomiting:** (unexplained vomiting, often associated with abdominal pain)	0	1	2	3	4	5		
**Rectal bleeding:** (bleeding from the bottom when passing a stool, fresh blood)	0	1	2	3	4	5		
**Abdominal pain:** (affects daily functioning such as school and sleep)	0	1	2	3	4	5		
**Wind/flatus:** (excessive burping and painful wind)	0	1	2	3	4	5		
**Bloating:** (bloated/extended stomach that is hard when pressed)	0	1	2	3	4	5		
**Screaming/back arching:** (screaming associated with back arching and kicking legs out straight)	0	1	2	3	4	5		
**Food aversion:** (pushes away food, gags, holds food in mouth, spits/throws food, cries during meals)	0	1	2	3	4	5		
